# 4-[3,4-Dimethyl-1-(4-methyl­phen­yl)-5-oxo-4,5-dihydro-1*H*-pyrazol-4-yl]-3,4-dimethyl-1-(4-methyl­phen­yl)-4,5-dihydro-1*H*-pyrazol-5-one

**DOI:** 10.1107/S1600536812009208

**Published:** 2012-03-10

**Authors:** Solange M. S. V. Wardell, Alan H. Howie, Edward R. T. Tiekink, James L. Wardell

**Affiliations:** a CHEMSOL, 1 Harcourt Road, Aberdeen, AB15 5NY, Scotland; b Department of Chemistry, University of Aberdeen, Meston Walk, Old Aberdeen, AB24 3UE, Scotland; c Department of Chemistry, University of Malaya, 50603 Kuala Lumpur, Malaysia; d Centro de Desenvolvimento Tecnológico em Saúde (CDTS), Fundação Oswaldo Cruz (FIOCRUZ), Casa Amarela, Campus de Manguinhos, Av. Brasil 4365, 21040-900, Rio de Janeiro, RJ, Brazil

## Abstract

In the title compound, C_24_H_26_N_4_O_2_, the complete mol­ecule is generated by the application of twofold symmetry. The pyrazole ring is approximately planar [r.m.s. deviation = 0.026 Å] and the benzene ring is twisted out of this plane [dihedral angle = 21.94 (7)°]. A twist in the mol­ecule about the central C—C bond [1.566 (3) Å] is also evident [C—C—C—C torsion angle = 44.30 (14)°]. Supra­molecular layers in the *bc* plane are formed in the crystal packing *via* C—H⋯O and C—H⋯π inter­actions.

## Related literature

For the therapeutic importance of pyrazole compounds, see: Sil *et al.* (2005 ▶); Haddad *et al.* (2004 ▶). For the diverse pharmacological activities of pyrazole compounds, see: Bekhit *et al.* (2010 ▶, 2012 ▶); Higashi *et al.* (2006 ▶). For synthetic background, see: Nef (1891 ▶): Veibel & Westöö (1953 ▶); Katritzky *et al.* (1997 ▶); Wardell *et al.* (2007 ▶); de Lima *et al.* (2010 ▶). For the synthesis of the title compound, see: Bernstein *et al.* (1947 ▶); Gryazeva & Golomolzin (2003 ▶).
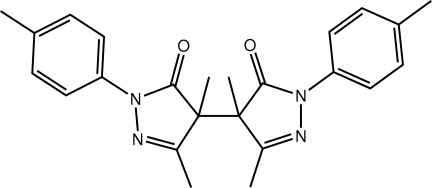


## Experimental

### 

#### Crystal data

C_24_H_26_N_4_O_2_
*M* *_r_* = 402.50
Monoclinic, 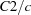

*a* = 23.0007 (8) Å
*b* = 6.6712 (2) Å
*c* = 13.5967 (5) Å
β = 92.566 (2)°
*V* = 2084.22 (12) Å^3^
*Z* = 4
Mo *K*α radiation
μ = 0.08 mm^−1^
*T* = 120 K
0.48 × 0.36 × 0.18 mm

#### Data collection

Rigaku Saturn724+ diffractometer
Absorption correction: multi-scan (*CrystalClear-SM Expert*; Rigaku, 2011 ▶) *T*_min_ = 0.668, *T*_max_ = 0.746
11383 measured reflections
2384 independent reflections
1856 reflections with *I* > 2σ(*I*)
*R*_int_ = 0.042

#### Refinement

*R*[*F* ^2^ > 2σ(*F* ^2^)] = 0.042
*wR*(*F* ^2^) = 0.136
*S* = 0.83
2384 reflections
139 parameters
H-atom parameters constrained
Δρ_max_ = 0.26 e Å^−3^
Δρ_min_ = −0.18 e Å^−3^

### 

Data collection: *CrystalClear-SM Expert* (Rigaku, 2011 ▶); cell refinement: *CrystalClear-SM Expert*; data reduction: *CrystalClear-SM Expert*; program(s) used to solve structure: *SHELXS97* (Sheldrick, 2008 ▶); program(s) used to refine structure: *SHELXL97* (Sheldrick, 2008 ▶); molecular graphics: *ORTEP-3* (Farrugia, 1997 ▶) and *DIAMOND* (Brandenburg, 2006 ▶); software used to prepare material for publication: *publCIF* (Westrip, 2010 ▶).

## Supplementary Material

Crystal structure: contains datablock(s) global, I. DOI: 10.1107/S1600536812009208/hg5185sup1.cif

Structure factors: contains datablock(s) I. DOI: 10.1107/S1600536812009208/hg5185Isup2.hkl

Supplementary material file. DOI: 10.1107/S1600536812009208/hg5185Isup3.cml

Additional supplementary materials:  crystallographic information; 3D view; checkCIF report

## Figures and Tables

**Table 1:** Hydrogen-bond geometry (Å, °) *Cg*1 is the centroid of the C6–C11 ring.

*D*—H⋯*A*	*D*—H	H⋯*A*	*D*⋯*A*	*D*—H⋯*A*
C4—H4*B*⋯O1^i^	0.98	2.60	3.5676 (16)	169
C4—H4*A*⋯*Cg*1^ii^	0.98	2.82	3.6644 (15)	145

Symmetry codes: (i) 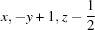; (ii) 
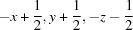
.

**Fig. 1.**
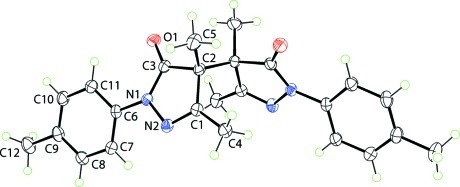
The molecular structure of (I) showing the atom-labelling scheme and displacement ellipsoids at the 50% probability level. Unlabelled atoms are related by the symmetry operation -x, y, 3/2 - z.

**Fig. 2.**
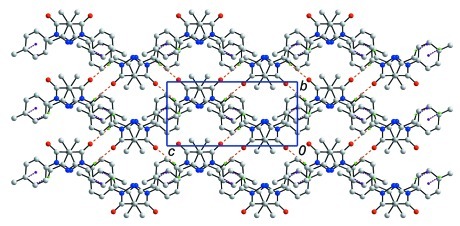
A view of the supramolecular layer in the bc plane of (I). The C—H···O and C—H···π interactions are shown as orange and purple dashed lines, respectively. Hydrogen atoms not involved in intermolecular interactions have been omitted for reasons of clarity.

**Fig. 3.**
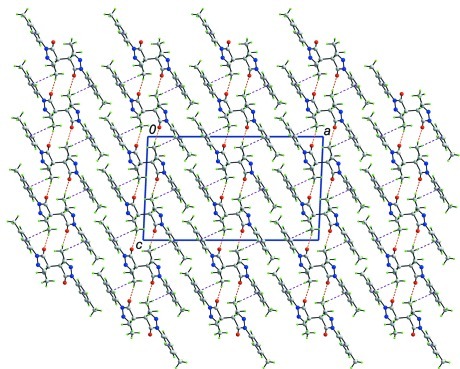
A view in projection down the b axis of the stacking of supramolecular layers along the a direction in (I). The C—H···O and C—H···π interactions are shown as orange and purple dashed lines, respectively.

## supplementary crystallographic information

### Comment

Pyrazoles are key structures in numerous compounds of therapeutic importance (Sil *et al.*, 2005, Haddad *et al.*, 2004). Compounds containing this ring system are known to display diverse pharmacological activities, for example as anti-malarial agents (Bekhit *et al.*, 2012), anti-inflammatory agents (Bekhit *et al.*, 2010), and against cardiovascular disease (Higashi *et al.*, 2006). A general route to pyrazole derivatives involves reaction of an arylhydrazine, ArNHNH_2_, with a β-dicarbonyl compound, *R*'COCH_2_COY. This reaction provides initially a hydrazone derivative, *R*NHN=C*R*'CH_2_COY, which can be isolated but which readily undergoes cyclization to a pyrazone derivative (Nef, 1891; Katritzky *et al.*, 1997; Wardell *et al.*, 2007; de Lima *et al.*, 2010). However, in some cases (Veibel & Westöö, 1953), a dimeric oxidation product is isolated, as found in the reaction between 4-MeC_6_H_4_NHNH_2_ and MeCOCH_2_CO_2_Et. The structure of this product, 4-[3,4-dimethyl-1-(4-methylphenyl)-5-oxopyrazol-4- yl]-4,5-dimethyl-2-(4-methylphenyl)pyrazol-3-one (I), is now reported.

The molecule of (I), Fig. 1, has crystallographically imposed twofold symmetry. The pyrazole ring is planar with a r.m.s. deviation for the fitted atoms of 0.026 Å; the maximum deviations from this plane are 0.019 (1) Å (for the N1 and C2 atoms) and -0.023 (1) Å (C3). The benzene ring is inclined to this plane forming a dihedral angle of 21.94 (7)°. There is a twist in the molecule about the central C—C bond [1.566 (3) Å] with the C1—C2—C2^i^—C1^i^ torsion angle being 44.30 (14)°; symmetry operation *i*: -*x*, *y*, 3/2 - *z*. The dihedral angle between the pyrazole rings is 61.78 (4)°.

In the crystal packing, supramolecular layers in the *bc* plane are formed by C—H···O and C—H···π interactions, Fig. 2 and Table 1. These stack along the *a* axis with no specific intermolecular interactions between them, Fig. 3.

### Experimental

A solution of 4-MeC_6_H_4_NHNH_2_.HCl (2 mmol) and MeCOCH_2_CO_2_Et (2 mmol) in EtOH (2 0 ml) was refluxed for 2 h. The reaction was left to slowly evaporate in air. Crystals were collected after a week, *M*.pt: > 573 K; lit. *M*.pt: >573 K (Bernstein *et al.*, 1947; Gryazeva & Golomolzin, 2003). IR ν: 3391, 3084, 3041, 3012, 2974, 2920, 2858, 1706, 1663, 1614, 1511, 1441, 1390, 1363, 1288, 1140, 1083, 1004, 912, 816, 776, 654, 590, 507, 485 cm^-1^.

### Refinement

The C-bound H atoms were geometrically placed (C—H = 0.95–0.98 Å) and refined as riding with *U*_iso_(H) = 1.2–1.5*U*_eq_(C). Owing to poor agreement two reflections, *i.e*. (5 1 2) and (2 0 2), were omitted from the final cycles of refinement.

### Crystal data

**Table:**

C_24_H_26_N_4_O_2_	*F*(000) = 856
*M**_r_* = 402.50	*D*_x_ = 1.283 Mg m^−^^3^
Monoclinic, *C*2/*c*	Mo *K*α radiation, λ = 0.71073 Å
Hall symbol: -C 2yc	Cell parameters from 6506 reflections
*a* = 23.0007 (8) Å	θ = 2.9–27.5°
*b* = 6.6712 (2) Å	µ = 0.08 mm^−^^1^
*c* = 13.5967 (5) Å	*T* = 120 K
β = 92.566 (2)°	Block, light-yellow
*V* = 2084.22 (12) Å^3^	0.48 × 0.36 × 0.18 mm
*Z* = 4	

### Data collection

**Table:**

Rigaku Saturn724+ diffractometer	2384 independent reflections
Radiation source: Rotating Anode	1856 reflections with *I* > 2σ(*I*)
Confocal monochromator	*R*_int_ = 0.042
Detector resolution: 28.5714 pixels mm^-1^	θ_max_ = 27.5°, θ_min_ = 3.0°
profile data from ω–scans	*h* = −29→29
Absorption correction: multi-scan (*CrystalClear-SM Expert*; Rigaku, 2011)	*k* = −8→8
*T*_min_ = 0.668, *T*_max_ = 0.746	*l* = −17→17
11383 measured reflections	

### Refinement

**Table:**

Refinement on *F*^2^	Primary atom site location: structure-invariant direct methods
Least-squares matrix: full	Secondary atom site location: difference Fourier map
*R*[*F*^2^ > 2σ(*F*^2^)] = 0.042	Hydrogen site location: inferred from neighbouring sites
*wR*(*F*^2^) = 0.136	H-atom parameters constrained
*S* = 0.83	*w* = 1/[σ^2^(*F*_o_^2^) + (0.104*P*)^2^ + 1.4411*P*] where *P* = (*F*_o_^2^ + 2*F*_c_^2^)/3
2384 reflections	(Δ/σ)_max_ < 0.001
139 parameters	Δρ_max_ = 0.26 e Å^−^^3^
0 restraints	Δρ_min_ = −0.18 e Å^−^^3^

### Special details

**Table:**

Geometry. All s.u.'s (except the s.u. in the dihedral angle between two l.s. planes) are estimated using the full covariance matrix. The cell s.u.'s are taken into account individually in the estimation of s.u.'s in distances, angles and torsion angles; correlations between s.u.'s in cell parameters are only used when they are defined by crystal symmetry. An approximate (isotropic) treatment of cell s.u.'s is used for estimating s.u.'s involving l.s. planes.
Refinement. Refinement of *F*^2^ against ALL reflections. The weighted *R*-factor *wR* and goodness of fit *S* are based on *F*^2^, conventional *R*-factors *R* are based on *F*, with *F* set to zero for negative *F*^2^. The threshold expression of *F*^2^ > 2σ(*F*^2^) is used only for calculating *R*-factors(gt) *etc*. and is not relevant to the choice of reflections for refinement. *R*-factors based on *F*^2^ are statistically about twice as large as those based on *F*, and *R*- factors based on ALL data will be even larger.

### Fractional atomic coordinates and isotropic or equivalent isotropic displacement parameters (Å^2^)

**Table:**

	*x*	*y*	*z*	*U*_iso_*/*U*_eq_	
O1	0.06343 (4)	0.52970 (14)	0.91141 (7)	0.0292 (3)	
N1	0.10162 (5)	0.23866 (16)	0.84739 (8)	0.0202 (3)	
N2	0.09381 (5)	0.12079 (17)	0.76120 (8)	0.0217 (3)	
C1	0.05610 (5)	0.2093 (2)	0.70361 (9)	0.0194 (3)	
C2	0.03383 (5)	0.40353 (18)	0.74559 (9)	0.0197 (3)	
C3	0.06614 (6)	0.40447 (19)	0.84662 (9)	0.0205 (3)	
C4	0.03921 (6)	0.1236 (2)	0.60511 (9)	0.0253 (3)	
H4A	0.0658	0.0144	0.5900	0.038*	
H4B	0.0413	0.2284	0.5549	0.038*	
H4C	−0.0006	0.0718	0.6057	0.038*	
C5	0.05690 (7)	0.5851 (2)	0.68862 (11)	0.0310 (3)	
H5A	0.0993	0.5753	0.6856	0.046*	
H5B	0.0468	0.7092	0.7224	0.046*	
H5C	0.0392	0.5861	0.6217	0.046*	
C6	0.13732 (5)	0.1626 (2)	0.92676 (9)	0.0198 (3)	
C7	0.15049 (6)	−0.0403 (2)	0.93131 (10)	0.0245 (3)	
H7	0.1366	−0.1285	0.8807	0.029*	
C8	0.18434 (6)	−0.1132 (2)	1.01097 (10)	0.0271 (3)	
H8	0.1931	−0.2523	1.0140	0.033*	
C9	0.20565 (6)	0.0114 (2)	1.08617 (9)	0.0258 (3)	
C10	0.19226 (6)	0.2144 (2)	1.07910 (10)	0.0282 (3)	
H10	0.2065	0.3028	1.1294	0.034*	
C11	0.15862 (6)	0.2913 (2)	1.00071 (10)	0.0253 (3)	
H11	0.1502	0.4306	0.9974	0.030*	
C12	0.24118 (7)	−0.0698 (3)	1.17294 (10)	0.0350 (4)	
H12A	0.2499	−0.2116	1.1618	0.052*	
H12B	0.2191	−0.0564	1.2326	0.052*	
H12C	0.2776	0.0057	1.1809	0.052*	

### Atomic displacement parameters (Å^2^)

**Table:**

	*U*^11^	*U*^22^	*U*^33^	*U*^12^	*U*^13^	*U*^23^
O1	0.0374 (6)	0.0238 (5)	0.0256 (5)	0.0034 (4)	−0.0081 (4)	−0.0085 (4)
N1	0.0224 (6)	0.0217 (5)	0.0161 (5)	0.0010 (4)	−0.0031 (4)	−0.0038 (4)
N2	0.0221 (6)	0.0267 (6)	0.0163 (5)	0.0006 (4)	−0.0001 (4)	−0.0047 (4)
C1	0.0198 (6)	0.0233 (6)	0.0153 (6)	−0.0011 (5)	0.0025 (5)	−0.0007 (5)
C2	0.0227 (7)	0.0197 (6)	0.0163 (6)	−0.0015 (5)	−0.0021 (5)	0.0008 (5)
C3	0.0217 (6)	0.0210 (6)	0.0184 (6)	−0.0028 (5)	−0.0010 (5)	−0.0002 (5)
C4	0.0276 (7)	0.0312 (7)	0.0170 (6)	0.0030 (6)	0.0002 (5)	−0.0037 (5)
C5	0.0361 (8)	0.0295 (8)	0.0269 (7)	−0.0107 (6)	−0.0036 (6)	0.0081 (6)
C6	0.0166 (6)	0.0260 (7)	0.0167 (6)	−0.0003 (5)	0.0004 (5)	0.0003 (5)
C7	0.0237 (7)	0.0250 (7)	0.0242 (7)	−0.0012 (5)	−0.0036 (5)	−0.0025 (5)
C8	0.0257 (7)	0.0262 (7)	0.0290 (7)	0.0027 (6)	−0.0025 (6)	0.0026 (5)
C9	0.0220 (7)	0.0363 (8)	0.0191 (6)	0.0025 (6)	0.0005 (5)	0.0023 (5)
C10	0.0266 (7)	0.0350 (8)	0.0224 (7)	0.0018 (6)	−0.0051 (6)	−0.0068 (6)
C11	0.0270 (7)	0.0254 (7)	0.0232 (7)	0.0002 (6)	−0.0037 (5)	−0.0033 (5)
C12	0.0338 (8)	0.0464 (9)	0.0242 (7)	0.0091 (7)	−0.0054 (6)	0.0028 (6)

### Geometric parameters (Å, º)

**Table:**

O1—C3	1.2177 (15)	C5—H5C	0.9800
N1—C3	1.3743 (17)	C6—C7	1.3882 (19)
N1—N2	1.4160 (14)	C6—C11	1.3943 (18)
N1—C6	1.4202 (16)	C7—C8	1.3928 (18)
N2—C1	1.2859 (17)	C7—H7	0.9500
C1—C4	1.4917 (17)	C8—C9	1.390 (2)
C1—C2	1.5142 (17)	C8—H8	0.9500
C2—C3	1.5323 (17)	C9—C10	1.391 (2)
C2—C5	1.5446 (18)	C9—C12	1.5057 (19)
C2—C2^i^	1.566 (3)	C10—C11	1.3873 (19)
C4—H4A	0.9800	C10—H10	0.9500
C4—H4B	0.9800	C11—H11	0.9500
C4—H4C	0.9800	C12—H12A	0.9800
C5—H5A	0.9800	C12—H12B	0.9800
C5—H5B	0.9800	C12—H12C	0.9800
			
C3—N1—N2	112.79 (10)	H5A—C5—H5C	109.5
C3—N1—C6	128.04 (11)	H5B—C5—H5C	109.5
N2—N1—C6	118.56 (10)	C7—C6—C11	119.98 (12)
C1—N2—N1	107.82 (10)	C7—C6—N1	119.99 (12)
N2—C1—C4	120.81 (12)	C11—C6—N1	120.03 (12)
N2—C1—C2	113.19 (11)	C6—C7—C8	119.23 (13)
C4—C1—C2	125.98 (11)	C6—C7—H7	120.4
C1—C2—C3	100.53 (10)	C8—C7—H7	120.4
C1—C2—C5	110.64 (10)	C9—C8—C7	121.98 (13)
C3—C2—C5	106.41 (10)	C9—C8—H8	119.0
C1—C2—C2^i^	112.51 (8)	C7—C8—H8	119.0
C3—C2—C2^i^	112.02 (12)	C8—C9—C10	117.50 (13)
C5—C2—C2^i^	113.78 (9)	C8—C9—C12	121.48 (14)
O1—C3—N1	126.61 (12)	C10—C9—C12	121.02 (13)
O1—C3—C2	127.80 (12)	C11—C10—C9	121.84 (13)
N1—C3—C2	105.52 (10)	C11—C10—H10	119.1
C1—C4—H4A	109.5	C9—C10—H10	119.1
C1—C4—H4B	109.5	C10—C11—C6	119.47 (13)
H4A—C4—H4B	109.5	C10—C11—H11	120.3
C1—C4—H4C	109.5	C6—C11—H11	120.3
H4A—C4—H4C	109.5	C9—C12—H12A	109.5
H4B—C4—H4C	109.5	C9—C12—H12B	109.5
C2—C5—H5A	109.5	H12A—C12—H12B	109.5
C2—C5—H5B	109.5	C9—C12—H12C	109.5
H5A—C5—H5B	109.5	H12A—C12—H12C	109.5
C2—C5—H5C	109.5	H12B—C12—H12C	109.5
			
C3—N1—N2—C1	2.35 (15)	C1—C2—C3—N1	3.64 (12)
C6—N1—N2—C1	174.14 (10)	C5—C2—C3—N1	−111.74 (12)
N1—N2—C1—C4	178.79 (11)	C2^i^—C2—C3—N1	123.33 (8)
N1—N2—C1—C2	0.36 (14)	C3—N1—C6—C7	152.61 (13)
N2—C1—C2—C3	−2.51 (13)	N2—N1—C6—C7	−17.78 (17)
C4—C1—C2—C3	179.15 (12)	C3—N1—C6—C11	−26.66 (19)
N2—C1—C2—C5	109.65 (13)	N2—N1—C6—C11	162.95 (11)
C4—C1—C2—C5	−68.69 (16)	C11—C6—C7—C8	0.90 (19)
N2—C1—C2—C2^i^	−121.85 (13)	N1—C6—C7—C8	−178.37 (11)
C4—C1—C2—C2^i^	59.82 (17)	C6—C7—C8—C9	−0.3 (2)
N2—N1—C3—O1	179.21 (12)	C7—C8—C9—C10	−0.4 (2)
C6—N1—C3—O1	8.4 (2)	C7—C8—C9—C12	178.74 (12)
N2—N1—C3—C2	−3.87 (13)	C8—C9—C10—C11	0.4 (2)
C6—N1—C3—C2	−174.71 (11)	C12—C9—C10—C11	−178.70 (13)
C1—C2—C3—O1	−179.48 (13)	C9—C10—C11—C6	0.2 (2)
C5—C2—C3—O1	65.14 (17)	C7—C6—C11—C10	−0.9 (2)
C2^i^—C2—C3—O1	−59.80 (14)	N1—C6—C11—C10	178.41 (11)

Symmetry code: (i) −*x*, *y*, −*z*+3/2.

### Hydrogen-bond geometry (Å, º)

Cg1 is the centroid of the C6–C11 ring.

**Table:**

*D*—H···*A*	*D*—H	H···*A*	*D*···*A*	*D*—H···*A*
C4—H4*B*···O1^ii^	0.98	2.60	3.5676 (16)	169
C4—H4*A*···*Cg*1^iii^	0.98	2.82	3.6644 (15)	145

Symmetry codes: (ii) *x*, −*y*+1, *z*−1/2; (iii) −*x*+1/2, *y*+1/2, −*z*−1/2.
